# Methylated site display (MSD)-AFLP, a sensitive and affordable method for analysis of CpG methylation profiles

**DOI:** 10.1186/s12867-017-0083-2

**Published:** 2017-03-09

**Authors:** Toshiki Aiba, Toshiyuki Saito, Akiko Hayashi, Shinji Sato, Harunobu Yunokawa, Toru Maruyama, Wataru Fujibuchi, Hisaka Kurita, Chiharu Tohyama, Seiichiroh Ohsako

**Affiliations:** 1 Laboratory of Environmental Health Sciences, Center for Disease Biology and Integrative Medicine, Graduate School of Medicine, The University of Tokyo, 7-3-1 Hongo, Bunkyo-ku, Tokyo, 113-0033 Japan; 2 Department of Radiation Effects Research, National Institutes for Quantum and Radiological Science and Technology, 4-9-1 Anagawa, Inage-ku, Chiba, 263-8555 Japan; 3 Maze, Inc., 1-2-17 Sennincho, Hachioji-shi, Tokyo, 193-0835 Japan; 4 Center for iPS Cell Research and Application, Kyoto University, 53 Kawahara-cho, Shogoin, Sakyo-ku, Kyoto, 606-8507 Japan; 5 Department of Life Science & Medical Bioscience, Graduate School of Advanced Science & Engineering, Waseda University, Tokyo, Japan; 6 Laboratory of Medical Therapeutics and Molecular Therapeutics, Gifu Pharmaceutical University, Gifu, Japan; 7 Faculty of Medicine, University of Tsukuba, Tsukuba, Japan

**Keywords:** DNA methylation profiling, AFLP, Epigenetics

## Abstract

**Background:**

It has been pointed out that environmental factors or chemicals can cause diseases that are developmental in origin. To detect abnormal epigenetic alterations in DNA methylation, convenient and cost-effective methods are required for such research, in which multiple samples are processed simultaneously. We here present methylated site display (MSD), a unique technique for the preparation of DNA libraries. By combining it with amplified fragment length polymorphism (AFLP) analysis, we developed a new method, MSD-AFLP.

**Results:**

Methylated site display libraries consist of only DNAs derived from DNA fragments that are CpG methylated at the 5′ end in the original genomic DNA sample. To test the effectiveness of this method, CpG methylation levels in liver, kidney, and hippocampal tissues of mice were compared to examine if MSD-AFLP can detect subtle differences in the levels of tissue-specific differentially methylated CpGs. As a result, many CpG sites suspected to be tissue-specific differentially methylated were detected. Nucleotide sequences adjacent to these methyl-CpG sites were identified and we determined the methylation level by methylation-sensitive restriction endonuclease (MSRE)-PCR analysis to confirm the accuracy of AFLP analysis. The differences of the methylation level among tissues were almost identical among these methods. By MSD-AFLP analysis, we detected many CpGs showing less than 5% statistically significant tissue-specific difference and less than 10% degree of variability. Additionally, MSD-AFLP analysis could be used to identify CpG methylation sites in other organisms including humans.

**Conclusion:**

MSD-AFLP analysis can potentially be used to measure slight changes in CpG methylation level. Regarding the remarkable precision, sensitivity, and throughput of MSD-AFLP analysis studies, this method will be advantageous in a variety of epigenetics-based research.

**Electronic supplementary material:**

The online version of this article (doi:10.1186/s12867-017-0083-2) contains supplementary material, which is available to authorized users.

## Background

In recent years, CpG methylation analyses have been focused mainly on epigenetics, allowing researchers to quantitatively assess important markers of differential gene expression. In particular, analyses by next-generation sequencing (NGS) provide extremely high-coverage genome-wide methylome data with all CpG methylation levels precisely measured [1, 2]. However, some of the whole-genome analyses are occasionally considered to be insufficient in terms of quantitative performance [3]. Moreover, the whole-genome methods remain unsuitable for investigations with large sample sizes owing to high costs. Nevertheless, a few genome-wide methods that can be performed at a relatively low cost per sample are available. For example, the Infinium Beadchip system, which is based on microarray technology and sodium bisulfite treatment, has recently been used for a large set of human blood DNA samples in massive cohort projects [4]. However, a major limitation is that the Infinium platform is designed only for CpG islands of the human genome [5, 6]. Therefore, alternative methods that can be better applied to large sample sizes should be developed. Furthermore, such a method should be convenient, cost-effective, and capable of processing multiple samples simultaneously, allowing small variations to be detected with adequate accuracy.

In this study we developed a technique, methylated site display (MSD), which displays only DNA fragments that are CpG-methylated at the 5′ end in the original genomic DNA sample. In combination with amplified fragment length polymorphism (AFLP) analysis [7–11], we designed MSD-AFLP analysis for obtaining methylated-CpGs profiles at a relatively low cost. By MSD-AFLP analysis, we compared the DNA methylation levels in three tissues from C57BL/6J mice to evaluate the precision and sensitivity of this method.

## Results

### Conceptual design of MSD-AFLP

**Fig. 1:**
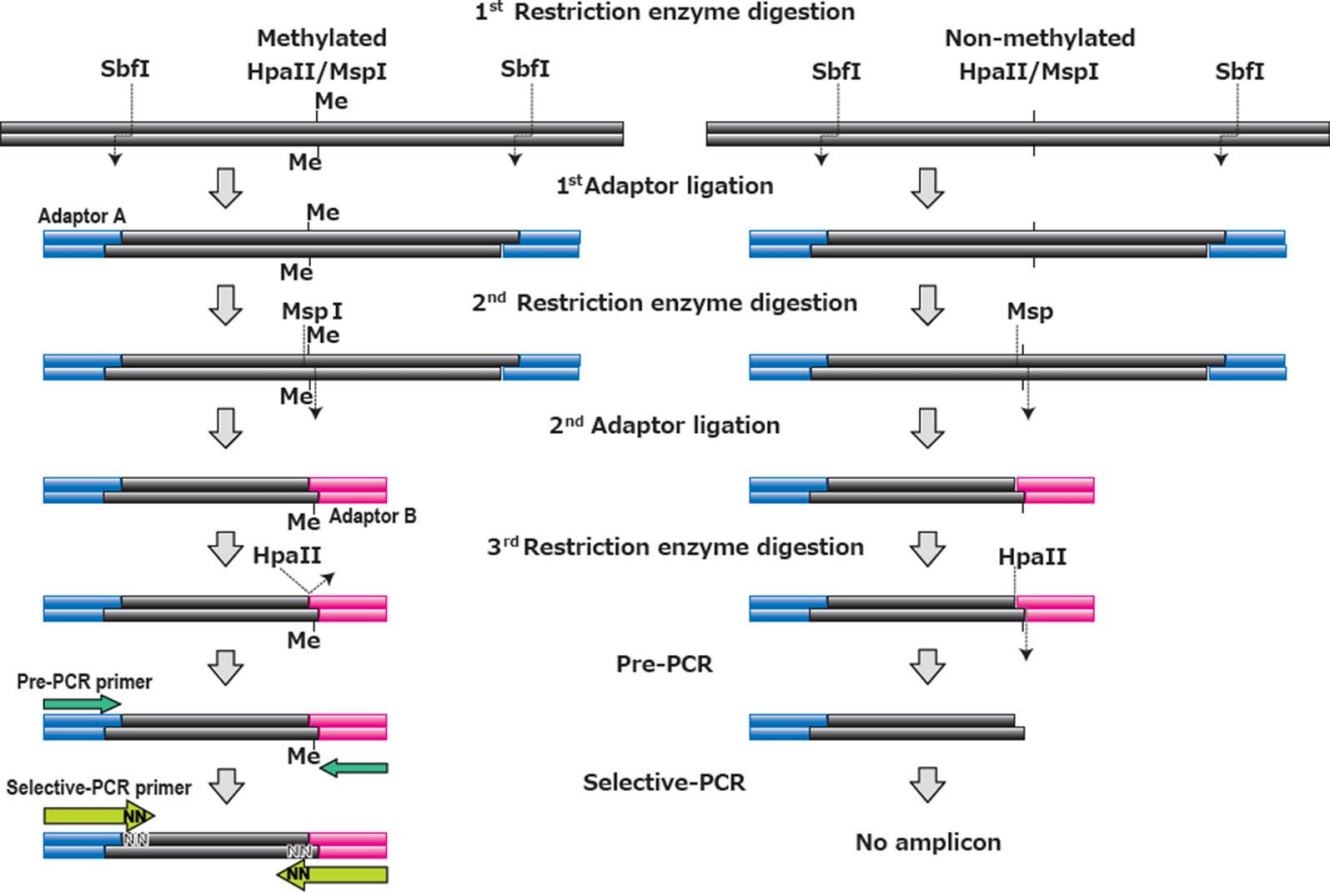
Flowchart of MSD-library preparation. Genomic DNA (100 ng) was digested with 10 units of the primary restriction enzyme *Sbf*I for 1 h and then ligated with 0.5 nmol Adaptor A using 400 units of T4 DNA ligase for 2 h. The treated sample was then digested with 100 units of the non-methylation-sensitive restriction enzyme *Msp*I (100 units) followed by ligation of the ends of the DNA fragment with Adaptor B. The ligated DNA fragments were then digested with 50 units of *Hpa*II for 1 h. Owing to the methylation sensitivity of *Hap*II, only DNA fragments with a methylated CpG retained Adaptor B, which was removed from all other fragments. The DNA fragments were then subjected to Pre-PCR using specific primers for Adaptor A and Adaptor B. Fragments that did not contain Adaptor B at this stage were not amplified. The Pre-PCR amplicons (MSD library) were then amplified as a subpopulation by selective-PCR with 6-carboxyfluorescein (6-FAM)-labelled selective-PCR primers. Finally, the selective-PCR products were electrophoresed with a capillary sequencer and separated by length

As shown in Fig. 1, DNA samples ligated with Adaptor A were digested with *Msp*I, an isoschizomer for *Hpa*II. The other end of the DNA fragments was ligated with Adaptor B and then digested with the methylation-sensitive *Hpa*II. If the Adaptor B-ligated fragment contains a methylated CpG, Adaptor B is not removed in this step. A similar description of the sensitivity of the hemi-methylated DNA to *Hpa*II was seen in the recent literature [12]. Only these DNA fragments retaining Adaptor B are amplified by the subsequent Pre-PCR to generate the MSD library. Therefore, DNA fragments sandwiched between the primary restriction enzyme (*Sfb*I) site and the nearest *Hpa*II site are to be amplified only when the nearest *Hpa*II-CpG is methylated.

A total of 1,594,127 *Hpa*II sites are found in the mouse reference genome. To obtain reliable and high-resolution AFLP electropherograms, it is necessary to keep sufficient spacing between signal peaks. When separated in a capillary sequencer, the preferable number of peaks should be smaller than 1000 in one run. Using the search capabilities of Genome DNA Fragment Database (GFDB), three primary restriction enzymes, *Sbf*I, *Pac*I and *Swa*I, were found to provide desirable peak numbers. In this study, therefore, we chose *Sbf*I as the primary restriction enzyme. We then used GFDB to calculate the number of *Sbf*I-*Hpa*II fragments as well as the distribution of fragment size in the mouse reference genome sequence to assess AFLP resolution (Additional file 1: Figure S2). It is understood that the ability to interpret peak data diminishes as fragment lengths overlap. Nonetheless, we found that 40,386 of the 47,315 fragments (85.4%) do not overlap in size and are predicted to display a single peak on an AFLP chart. Despite covering only 0.22% of all CpGs in the reference genome (21,342,779 CpGs) in one analysis, this technique seems to have sufficient profiling capabilities. In addition, as a result of examining the distribution of methylated sites detected by this method, CpG sites in intragenic regions, which can be detected by MSD-AFLP, were 55.3% out of the whole genome.

We then expanded GFDB to apply other organisms, i.e., human (Additional file 1: Figure S2), zebrafish and *Neurospora crassa*. The number of *Sbf*I-*Hpa*II fragments as well as the distribution of fragment size in the human, zebrafish, and *N. crassa* reference genome sequences were used to assess AFLP resolution in the same way as in the mouse genome sequence. We found that 47,315 of the 56,799 fragments (75.0%) in humans and 20,006 of the 22,113 fragments (89.4%) in zebrafish do not overlap in size and are predicted to display a single peak on an AFLP chart. However, in the case of *N. crassa*, only appoximately 1000 *Sbf*I-*Hpa*II fragments were found, suggesting that *Sbf*I cuts *N. crassa* DNA much less than it does the other three organisms. Therefore, alternative restriction enzymes such as *Nco*I, *Ase*I, or *Bsp*HI should be used. We found that 18,139 of the 19,995 *Nco*I-*Hpa*II fragments (90.7%) do not overlap in size on an AFLP chart of *N. crassa*.

### Reproducibility of MSD-AFLP

**Fig. 2:**
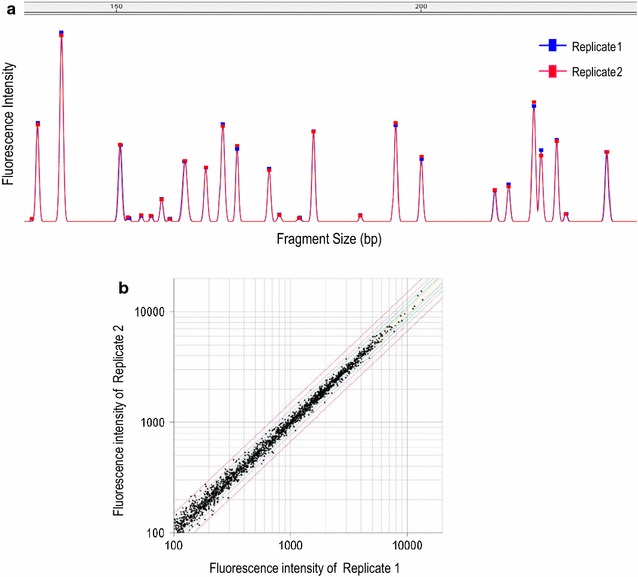
Reproducibility of MSD-AFLP analysis. **a** Two AFLP peak charts from MSD libraries independently generated from the same genomic DNA preparation are presented in this *panel* as Replicates 1 and 2. Profiles of the signal peaks from the two independent experiments were in high agreement. **b** *Scatter plots* of Replicate 1 and 2. *Red*, *pink*, *green*, *blue*, and *yellow lines* indicate a 1.5-, 1.4-, 1.3-, 1.2-, and 1.1-fold differences, respectively

We examined the reproducibility of MSD-AFLP by comparing two MSD libraries independently constructed from the same kidney DNA preparation. AFLP analysis was performed for each library using 16 selective primer sets, resulting in a total of 2003 signal peaks to be compared. We found that the methylation level profiles of the two experimental replicates coincided well with one another, as shown by the florescence peaks in Fig. 2a. The coefficient of determination, R^2^, was 0.992, indicating reliable reproducibility of MSD-AFLP (Fig. 2b).

### Accuracy of MSD-AFLP

**Fig. 3:**
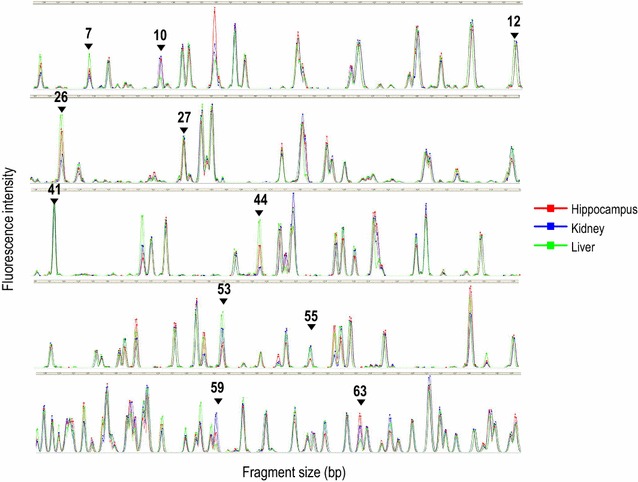
AFLP electropherogram peak charts obtained by MSD-AFLP analysis. Each color electropherogram represents data from one of three tissues: *Red* hippocampus; *blue* kidney; *green* liver. A total of 9 electropherograms are seen in the charts, because three samples from each tissue were analyzed. *Black* numbers and *arrows* indicate Peak IDs (11 CpGs) randomly selected for this study. Note that several CpGs were detected as differentially methylated across the three types of tissue, as seen in Peak ID 26, 44, and 53. Furthermore, among the three mouse samples, methylation patterns were in agreement among the tissues

Using MSD-AFLP, we compared the methylation levels of three mouse tissues (liver, kidney, and hippocampus). For each tissue, we used 16 selective primer sets out of 256 possible sets for PCR. We detected 2449 AFLP signals and succeeded in identifying CpG sites that are differentially methylated among the three kinds of tissue (Fig. 3). Eleven signal peaks were randomly selected and submitted as an inquiry to GFDB to retrieve candidate loci for the CpG sites. In parallel, the sequences of the 11 DNA fragments were directly determined by gel isolation. Although three extra false DNA loci were retrieved, all of the 11 DNA sequences matched the candidate loci predicted by GFDB (Additional file 1: Table S4). The percentage of one-to-one correspondence was 72.7% in this case. Additionally, we performed another 56 runs of gel isolation to determine the sequences. Out of them, the 45 sequences represented one-to-one correspondence (80.4%) (data not shown). These values are very reasonable considering the non-overlapping ratio (85.4%) predicted in Additional file 1: Figure S2B.

**Fig. 4:**
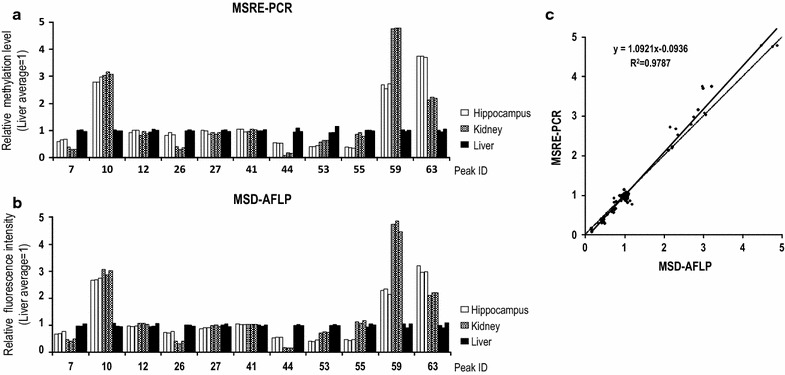
Confirmation of the accuracy of MSD-AFLP analysis by MSRE-PCR. **a** Relative methylation levels of 11 CpG sites in Fig. 3 determined by MSRE-PCR analysis using locus-specific primers. **b** Relative fluorescence intensity obtained from MSD-AFLP data. **c** *Scatter plot* of relative methylation level determined using MSRE-PCR analysis and relative fluorescence intensity determined using MSD-AFLP analysis. The *dashed line* indicates y = x whereas the *solid line* indicates the line of best fit. A high coefficient of correlation was determined (R^2^ = 0.9787)

Next, we designed locus-specific primers for MSRE-PCR analysis in accordance with the reference sequences of the 11 DNA fragments to measure the relative methylation levels of *Hpa*II-CpG sites and compare them with the relative fluorescence intensities obtained by MSD-AFLP analysis (Fig. 4a). MSD-AFLP analysis had showed relative values similar to that of the MSRE-PCR relative values in all 11 fragments, suggesting that the two are consistent (Fig. 4b). Furthermore, a scatter plot of the relative values of the two methods indicates a strong correlation between the two (R^2^ = 0.9787) (Fig. 4c).

### Percent methylation level from MSD-AFLP peak charts

**Fig. 5:**
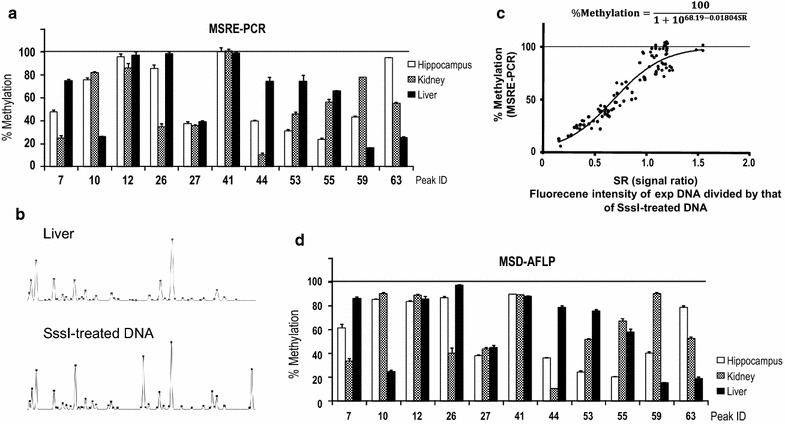
Percent methylation levels of CpGs in the MSD-AFLP peak charts. **a** Percent methylation levels derived from MSRE-PCR. **b** Typical peak chart from liver DNA (*upper*) and *Sss*I-treated artificially methylated DNA (*lower*). **c** *Scatter plot* of signal ratios (SR) and percent methylation levels (derived from MSRE-PCR) of 11 CpGs. SR was calculated by dividing the fluorescence intensities derived from MSD-AFLP by that of the *Sss*I-treated artificially methylated DNA. An approximation formula expressing the relationship between the percent methylation levels determined by MSRE-PCR analysis and SR was developed using the Hill equation. **d** Percent methylation levels of the MSD-AFLP peak charts were determined by substituting SR into the Hill equation

The percent methylation levels of each CpG were calculated from the fluorescence intensity of each peak in MSD-AFLP as follows. First, we determined the percent methylation levels of the 11 CpGs by MSRE-PCR analysis, as shown in Fig. 5a. Next, we subjected *Sss*I-treated artificially methylated DNA to MSD-AFLP analysis in order to theoretically obtain fully methylated signal intensities at each CpG site (Fig. 5b). Using the fluorescence intensities of the 11 peaks in Fig. 3, signal ratios (SR) were obtained by dividing the fluorescence intensity of each tissue DNA by that of the *Sss*I-treated artificially methylated DNA. After scatter plotting this data, an approximation formula expressing the relationship between the percent methylation determined by MSRE-PCR analysis and SR was developed using the Hill equation in order to normalize the values to a range from 0 to 100 (Fig. 5c). Finally, the percent methylation levels of all CpGs in the MSD-AFLP peak charts were calculated by substituting SR into this approximation formula. The methylation levels of the 11 CpGs are shown in Fig. 5d.

To further verify the percent methylation levels of the MSD-AFLP peak charts, we randomly selected two Peak IDs, 44 and 59, for bisulfite genomic sequencing for methylation analysis. Our results showed that the percent methylation levels obtained by MSD-AFLP analysis were highly consistent with those obtained by bisulfite genomic sequencing in the three tissues, as well as those by MSRE-PCR analysis (Additional file 1: Figure S3).

Finally, the percent methylation levels of all 2449 CpGs in the three tissues were analyzed by hierarchical clustering analysis and principal component analysis (PCA) (Additional file 1: Figure S4). Significant clusters were found for every tissue, highlighting the capability of MSD-AFLP analysis to detect unique and contrasting methylation patterns between tissues. Moreover, significant isolation of the principal of each tissue component was observable by PCA.

### Sensitivity of MSD-AFLP analysis

**Fig. 6:**
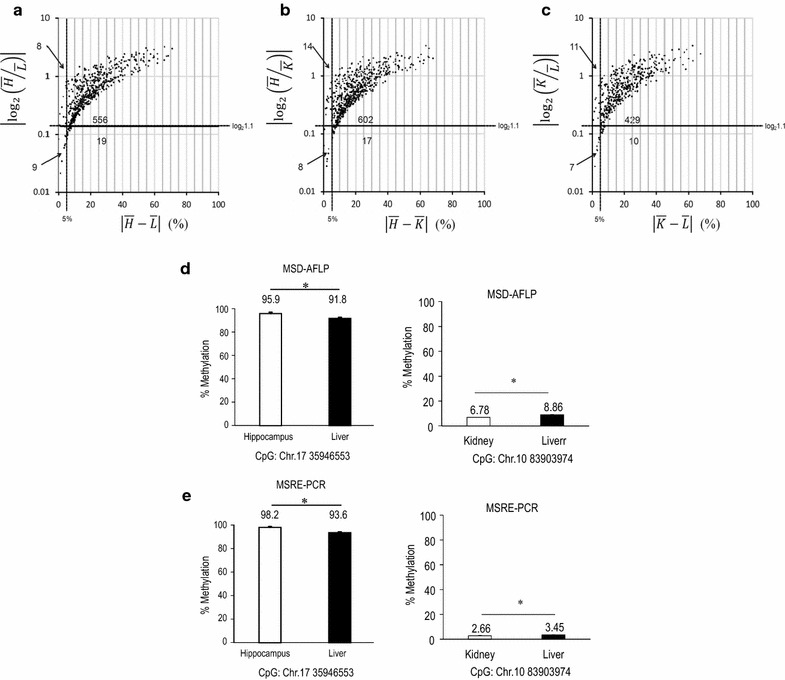
Sensitivity of MSD-AFLP analysis. **a**–**c** *Scatter* *plots* of CpGs where MSD-AFLP analysis showed statistically significant differences between two tissues as determined by one-way ANOVA using Benjamini–Hochberg corrections followed by the post hoc Tukey test. Tissue data is derived from the average of three mouse specimens. Significant differences in methylation level were found in the 592 CpGs between the hippocampus and the liver (**a**), 641 CpGs between the hippocampus and the kidney (**b**), and 457 CpGs between the kidney and the liver (**c**).* X*-axis, absolute values of the difference in percent methylation level;* y*-axis, absolute values of the ratio of percent methylation level. *H* hippocampus; *L* liver; *K* kidney. Note that **a**, 9 CpGs; **b**, 8 CpGs; and **c**, 7 CpGs were found to be statistically significant in both differences in percent methylation levels of less than 5% and ratios of percent methylation level of less than 1.1-fold. **d** Example of slight but statistically significant difference in percent methylation level (Chr.17 35946553 and Chr.10 83903974) between two tissues derived from MSD-AFLP data. Student’s *t* test. *p < 0.05. **e** Confirmation of statistically significant difference in methylation level **d** by analyzing the same CpG site using MSRE-PCR with locus-specific primers. Student’s *t*-test. *p < 0.05

In order to assess the sensitivity of MSD-AFLP analysis to subtle differences in methylation levels, we evaluated the differences in CpG-methylation levels between each tissue. By one-way ANOVA, we found that a total of 805 CpGs out of the analyzed 2449 CpGs have statistically significant differences in methylation level. We identified the combination of tissues responsible for this difference using the post hoc Tukey test. We found showed a statistically significant differences in methylation level in 592 CpGs between the hippocampus and the liver (Fig. 6a), in 641 CpGs between the hippocampus and the kidney (Fig. 6b), and in 457 CpGs between the kidney and the liver (Fig. 6c). Furthermore, our results indicated that MSD-AFLP analysis had the sensitivity to detect 24 CpGs with less than 5% and 1.1-fold differences in methylation levels and ratios of methylation levels, respectively. In Fig. 6d, we present two randomly selected CpGs, which have slight but statistically significant differences in methylation levels determined by MSD-AFLP analysis between the hippocampus and the liver (Chr.17 35946553), and the kidney and the liver (Chr.10 83903974). For confirmation, MSRE-PCR analysis using locus-specific primers for measuring percent methylation level also detected a similar statistically significant difference between the two tissues (Fig. 6e).

## Discussion

In this study, we developed a unique method, MSD-AFLP analysis, for determining CpG methylation level profiles with high sensitivity and accuracy. Although MSD-AFLP analysis covers only 0.22% of CpGs sites out of the whole genome, it can provide CpG methylation level profiles of a multitude of CpGs (approximately 40,000) in a single analysis with almost the same precision as MSRE-PCR analysis, a quantitative PCR method, as well as with relatively low cost compared with other current array-based or NGS-based genome wide DNA methylation analyses.

The widespread use of NGS technology has led to a number of methods for analyzing CpG methylation levels within the whole genome. Of these, whole-genome bisulfite sequencing is the most powerful technique, providing extremely high-coverage genome wide methylome data with all CpG methylation levels precisely measured [1, 2, 13]. Similarly, methylated DNA immunoprecipitation-seq [14] and *Hpa*II tiny fragment enrichment by ligation-mediated PCR-tagging [15, 16] analyses were developed by incorporating NGS. However, these methods remain unsuitable for investigations with large sample sizes on account of their expensiveness and do not offer satisfactory quantitative performance even when more expensive measures are taken to obtain sufficient depths. Reduced representation bisulfite sequencing can provide quantitative values of numerous CpG methylations [17] however, even in analyses utilizing machines such as SOLiD (Thermo Fisher Scientific, Inc., San Diego, CA, USA) and Hiseq 2000 (Illumina, Inc., Waltham, MA, USA), the average depth of coverage is usually only approximately 30–100 reads [18, 19]. Out of all current NGS technologies, only the Roche 454 sequencing system (Roche Diagnostics), which is capable of obtaining relatively long sequences in one read, can provide such a high rate of mapping. Even so, with the Roche 454 system, more than 1000 reads are required to detect a 5% methylation level difference in the sequence of one sample [20, 21]. In contrast, the MSD-AFLP analysis established in this study was capable of easily detecting significant differences of less than 5% in methylation level (Fig. 6). In current studies of methylation analyses, huge numbers of samples containing various cell types are usually required to obtain significant data [4]. Since multiple samples can be processed simultaneously in MSD-AFLP analysis, allowing small variations to be detected with adequate accuracy at a low cost, this method will be advantageous for a variety of epigenetics-based research studies.

Regarding the cost-benefit of current genome wide analyses, Infinium^®^ assay (HumanMethylation450 Beadchip) has become the preferred choice, which can be used to analyze the methylation levels of approximately 450,000 CpGs [5, 6]. At present, however, this platform is designed only for the human genome and is biased towards CpG islands in the promoter region. In contrast, MSD-AFLP analysis can be used for any kind of organism.

In the research fields of hygiene and environmental toxicology, it has been pointed out that environmental chemicals and pollutants can cause diseases that are developmental in origin, possibly resulting from abnormal epigenetic alterations such as those in DNA methylation [22]. Several genome wide DNA methylation analyses showed that environmental chemicals, such as vinclozolin and bisphenol-A, can cause changes in CpG methylation level, which can be transmitted to next-generation offspring [23–26]. These inheritable DNA methylation changes were measured using sperm nuclear DNA; however, the reliability and reproducibility of these studies are still controversial [27]. In terms of verifying the accuracy of previous reports, our MSD-AFLP analysis will be useful for analyzing such subtle changes in the CpG methylation pattern induced by environmental factors that are transmitted to later generations.

With regard to other applications, MSD-AFLP analysis will also be a useful tool in clinical cancer research. Investigating the epigenetic markers of cancer stem cells in a multitude of clinical samples is of significant interest [28–31]. Although the genome coverage of MSD-AFLP is 0.22% out of all CpG sites in the whole genome, MSD-AFLP analysis can be used to screen a large number of clinical samples with relatively low cost.

## Conclusion

MSD-AFLP analysis can be potentially used to measure slight changes in CpG methylation level. On the basis of our results regarding the remarkable precision, sensitivity, and throughput of MSD-AFLP, we conclude that this method will be advantageous in a variety of epigenetics-based studies.

## Methods

### Reagents

The reagents and materials used in this study were purchased from the manufacturers indicated in parentheses: CpG methyltransferase (*M.Sss*I), T4 DNA ligase, and restriction enzymes *Hpa*II, *Msp*I, *Sbf*I, and *Stu*I (New England Biolabs, MA, USA) it guarantees that the efficiency of their restriction enzymes is almost and the methylation of CpG blocks 100% *Hpa*II digestion reaction; EpiTect Bisulfite Kit and AllPrep DNA/RNA Mini Kit (Qiagen, Hilden, Germany); Oligonucleotides (Operon, Alameda, CA, USA); Magnetic beads coated with streptavidin (Dynabeads^®^ M-280 Streptavidin) (Dynal, Oslo, Norway); TITANIUM Taq DNA polymerase (Takara Bio, Kusatsu, Japan); GenElute™ Agarose Spin Columns (Sigma-Aldrich, St. Louis, MO, USA); Ligation Convenience Kit (Nippon Gene, Tokyo, Japan); pGEM^®^-T Easy Vector (Promega, Madison, WI, USA); Competent Cell DH5α and Insert Check-Ready (Toyobo, Osaka, Japan); LightCycler^®^ 480 SYBR Green I Master (Roche Diagnostics GmbH, Mannheim, Germany); POP-7™ Polymer, GeneScan™ 500 LIZ^®^ Size Standard, and BigDye^®^ Terminator v3.1 Cycle Sequencing Kit (ThermoFisher Scientific Inc., San Diego, CA, USA).

### Animals and tissues

Thirteen-week old male C57BL/6 J mice (n = 3) purchased from CLEA Japan Inc. (CLEA Japan Inc., Tokyo, Japan) were sacrificed by cervical dislocation to collect liver, kidney, and hippocampus samples.

### Artificially CpG-methylated genomic DNA

Genomic DNA was purified with the AllPrep DNA/RNA Mini Kit. To generate the artificially methylated DNA in all CpG sites, 2 μg of mouse kidney genomic DNA was incubated with S-adenosylmethionine and *Sss*I at 37 °C for 1 h and subsequently incubated at 65 °C for 20 min. The treated DNA was again purified with the AllPrep DNA/RNA Mini Kit. We confirmed the quality of the artificially methylated DNA by MSRE-PCR targeting on three randomly selected CpGs. The methylation levels of these CpGs were over 97%.

### MSD library

A flowchart of the MSD-library preparation steps is shown in Fig. 1. First, genomic DNA (100 ng) digested with *Sbf*I was ligated with a biotinylated adaptor (Adaptor A) using 400 units of T4 DNA ligase. Next, the ligated products were digested with 100 units of the methylation-insensitive enzyme *Msp*I for 1 h, an isoschizomer of methylation-sensitive *Hpa*II that recognizes and digests CCGG sequences. The resulting DNA fragments were captured using Dynabeads^®^ M-280 Streptavidin and washed with washing buffer (10 mM Tris HCl, 1 mM EDTA, 2 M NaCl, pH7.5) and TE (1 mM Tris HCl, 0.1 mM EDTA, pH7.5) three times. The DNA fragments were then ligated with Adaptor B. After another washing in the same manner, the products were digested with *Hpa*II on the magnetic beads. While remaining on the beads, the *Hpa*II-digested DNA fragments were then amplified with the Pre-PCR primers under the following conditions: 25 cycles of denaturation at 95 °C for 20 s, annealing at 58 °C for 20 s, and extension at 72 °C for 90 s. The resulting solution containing the MSD library was used as a template for selective PCR. All adaptors and primers used in MSD-library construction are listed in Additional file 1: Table S1.

### Selective-PCR and electrophoresis

The selective-PCR step in MSD-AFLP analysis is based on the original report on AFLP [8]. The set of selective-PCR primers is shown in Additional file 1: Table S1. We prepared 16 sequences each of the *Msp*I-NN primer and *Sbf*I-NN primer. The 5′ end of the *Msp*I-NN primer was labeled with 6-carboxyfluorescein (6-FAM). PCR was performed in a 10 μL solution containing 10 pmol of the *Msp*I-NN primer, 10 pmol of the *Sbf*I-NN primer, 40 nmol of dNTPs, and 0.2 μL of TITANIUM Taq DNA polymerase in accordance with the manufacturer’s instructions. The cycling conditions were as follows: first denaturation at 95 °C for 1 min and 28 cycles of denaturation at 95 °C for 20 s, annealing at 66 °C for 30 s, and extension at 72 °C for 2 min. The resultant PCR products were electrophoresed using an Applied Biosystems 3730xl DNA Analyzer (ThermoFisher Scientific). Data were analyzed using GeneMapper^®^ *ID* Software v3.7 (ThermoFisher Scientific) and HiAL version 5.2 software developed by Maze Inc. (Tokyo, Japan).

### DNA isolation and sequencing

The DNA of fragments was sequenced as follows. An aliquot of 1 μL of MSD-AFLP analysis product was separated on a denaturing polyacrylamide gel containing 7.0 M urea. Fluorescence from this product was detected using Typhoon 9210 Molecular Imager (Amersham Biosciences, Piscataway, NJ, USA) and slices of gel containing the DNA fragments were cut out. The gel slices were suspended in 50 μL of TE buffer with 1 μL of the suspension being used for PCR with *Msp*I-universal and *Sbf*I-universal primers (Additional file 1: Table S1). The DNA sequence of the PCR product was determined using the *Msp*I-universal primer and BigDye^®^ Terminator v3.1 Cycle Sequencing Kit.

### MSRE-PCR

Methylation-sensitive restriction enzyme dependent PCR (MSRE-PCR) was performed as follows. All locus-specific primers used in this experiment were designed to amplify the target DNA which has *Hpa*II-CpG (Additional file 1: Table S2). Purified genomic DNA (100 ng) was divided into two portions. One aliquot was digested with methylation-sensitive restriction enzyme *Hpa*II while the other aliquot was digested with *Stu*I. *Stu*I was selected as a restriction enzyme that does not cut any of the 11 target DNAs. The *Hpa*II- and StuI-digested DNAs were subjected to quantitative-PCR using a LightCycler^®^ 480. PCR was performed under the following conditions: 95 °C for 5 min and 50 cycles of 95 °C for 10 s, 63 °C for 20 s, and 72 °C for 10 s, followed by determination of the melting curve at 95 °C for 5 s, 65 °C for 1 min, and 97 °C for continuous hold. The methylation levels (expressed as % methylation) of *Hpa*II-CpG sites are presented here as a ratio of the target copy number from the *Hpa*II-digested DNA to that from the *Stu*I-digested DNA.

### Bisulfite genomic sequencing

Sodium bisulfite conversion and purification were performed using the EpiTect Bisulfite Kit. The bisulfite-treated DNA was amplified and purified using SIGMA GenElute. The purified DNA was cloned using the pGEM^®^-T Easy Vector with the Ligation Convenience Kit and transformed into DH5α. Colony PCR was performed to identify positive clones. Sequences were then determined using the BigDye^®^ Terminator v3.1 Cycle Sequencing Kit and the M13 reverse primer, GCGGATAACAATTTCACACAG. All primers used in this step are listed in Additional file 1: Table S3.

### Prediction of genomic position from AFLP peak charts

In order to predict the genomic position of methylated CpGs from AFLP peak charts, we developed the GFDB (Additional file 1: Figure S1, http://gfdb.maze.co.jp/). GFDB is composed of a versatile search interface and a virtual AFLP data generation system based on input reference genome sequences. GFDB can simulate the MSD-AFLP procedure of genomic DNA cleavage with any restriction enzyme or any selective PCR. Under a given condition, it shows the number of DNA fragments produced by selecting a combination of restriction enzymes, fragment length range, and two selective nucleotides adjacent to each desired recognition sequence (Additional file 1: Figure S1).

### Statistical analyses

Diffrences in methylation levels between the tissues were analyzed by one-way ANOVA followed by the post hoc Tukey test using R statistical software (http://cran.r-project.org/). Multiple comparison adjusted *p*-values were computed using Benjamini–Hochberg (BH) corrections [32]. Statistical probabilities of FDR ≦ 0.05 were considered significant. Using R, we normalized CpG methylation levels to the z-score and subjected to PCA and hierarchical clustering analysis of methylation pattern utilizing Euclidean distance and the unweighted pair-group method using arithmetic mean (UPGMA). Finally, an approximation formula derived from Hill equation was developed using GraphPad Prism (GraphPad Software, La Jolla, CA, USA).

## Additional file

**Additional file 1**: **Table S1.** Oligonucleotides used for MSD-AFLP. **Table S2.** Locus-specific primers used for MSRE-PCR. **Table S3.** Locus-specific primers used for bisulfite genomic sequencing. **Table S4.** Chromosomal nucleotide position of methylated cytosine predicted using the Genome DNA Fragment Database (GFDB) and gene name identified by actual sequencing analysis. **Figure S1.** Genome DNA Fragment Database (GFDB). **Figure S2.** Length data of *Sbf*I-*Hpa*II/*Msp*I fragments in the mouse and human reference genome sequences, retrieved using the GFDB system. **Figure S3.** Analysis with bisulfite genome sequencing. **Figure S4.** Comparison of genome wide methylation patterns among tissues using MSD-AFLP data.

## Abbreviations

MSD: methylated site display
AFLP: amplified fragment length polymorphism
PCR: polymerase chain reaction
qPCR: quantitative PCR
MSRE: methylation-sensitive restriction enzyme
