# Supplementary material for: Methylated site display (MSD)-AFLP, a sensitive and affordable method for analysis of CpG methylation profiles
Source: BMC Mol Biol. 2017 Mar 9;18:7. doi: 10.1186/s12867-017-0083-2 (PMC5345256; doi:10.1186/s12867-017-0083-2)
Supplement: Supplementary file 1 — Additional file 1: Table S1. Oligonucleotides used for MSD-AFLP. Table S2. Locus-specific primers used for MSRE-PCR. Table S3. Locus-specific primers used for bisulfite genomic sequencing. Table S4. Chromosomal nucleotide position of methylated cytosine predicted using the Genome DNA Fragment Database (GFDB) and gene name identified by actual sequencing analysis. Figure S1. Genome DNA Fragment Database (GFDB). Figure S2. Length data of SbfI-HpaII/MspI fragments in the mouse and human reference genome sequences, retrieved using the GFDB system. Figure S3. Analysis with bisulfite genome sequencing. Figure S4. Comparison of genome wide methylation patterns among tissues using MSD-AFLP data. [file 12867_2017_83_MOESM1_ESM.pdf]

Additional file 1

Table S1. Oligonucleotides used for MSD-AFLP

Oligo-name		Sequence (5' to 3')
Oligos for adaptors:		
*Sbf*I-adapter (Adapter A)	Upper	TCCGACTGGTATCAACGCAGAGTACTAGAGTTGCA
	Lower[a]	p-ACTCTAGTACTCTGCGTTGATACCAGTCGGA
*Msp*I-adapter (Adapter B)	Upper[b]	b-AATGGCTACACGAACTCGGTTCATGACC
	Lower[a]	p-CGTGTCATGAACCGAGTTCGTGTAGCCATT
Primers for MSD-AFLP:		
Pre-PCR primer	Forward	AATGGCTACACGAACTCGGTTCATGACACGG
	Reverse	TCCGACTGGTATCAACGCAGA
Selective-PCR primer	*Msp*I-NN primer[c]	f-AATGGCTACACGAACTCGGTTCATGACAIIINN
	*Sbf*I-NN primer	AGAGTACTAGAGTTGCAGGNN
*Msp*I-universal primer		AATGGCTACACGAACTCGGTTCATGACA
*Sbf*I-universal primer		AGAGTACTAGAGTTGCAGG

[a] p-, 5'-phosphorylation.

[b] b-, 5'-biotinylation.

[c] f-, 5'-6-carboxyfluorescein (6-FAM) conjugation.

Additional file 1

Table S2. Locus-specific primers used for MSRE-PCR

Peak ID	Locus		Sequence (5' to 3')	Size (bp)
7	Chr.3 83588206	Forward	AAGAGGATCGGTCCTAAAAA	155
		Reverse	GGGCTTTCAGGTTCTCC	
10	Chr.9 53410271	Forward	TGCTATACAAACAACTCGGTCAA	158
		Reverse	AGGGTTTGGCTGAACAAAAAT	
12	Chr.4 138720271	Forward	TGTGAGGGACAGAGAAACGAT	130
		Reverse	AACATTAGCAGGCAAACTGGA	
26	Chr.13 110215936	Forward	ACCAGCTACACGGCTCGTAAT	150
		Reverse	TAAAACGGGTGGAAGGAGATT	
27	Chr.2 30409726	Forward	ACAGTGTCACATTCCCTCCAG	185
		Reverse	ACCCCTGTCCTTCAGAACTGT	
41	Chr. 7 31402396	Forward	TGAGAATGCAGATACCCAAGG	197
		Reverse	CAGGTGACCCAAAAAGACAAA	
44	Chr. 7 29587740	Forward	GCTTCCAAACAGTAGAGCTTCC	179
		Reverse	CTCAGGACAAACCATGCAGA	
53	Chr.5 52443251	Forward	ACAAAAGCTGGCTGCATTCT	170
		Reverse	AGGAAGCTCGGAAATGACAAT	
55	Chr.11 76224631	Forward	GGCCCTTTTGAAATCAAGGT	150
		Reverse	GTTCTTCCAGTCCGACTTTCC	
59	Chr.10 67595674	Forward	TTTTGGGAACTTGAACCAGTG	149
		Reverse	TCTTCTGGAAGGTTTGCTGTG	
63	Chr.17 44272079	Forward	GCTAGAAAGCCAGGAGTACGAA	124
		Reverse	GGACTCTGAAAACACCTCATCC	
	Chr.17 35946553	Forward	GGAGGATTGCTGAGCACATAA	122
		Reverse	CCATGACTTCTCCAGAAAGGA	
	Chr.10 83903974	Forward	GCCACCCTTCTTCATGTTTG	127
		Reverse	GCTGGAGGGGACTGTGACT	

Table S3. Locus-specific primers used for bisulfite genomic sequencing

Peak ID	Locus		Sequence (5' to 3')
44	Chr. 7 29587740	Forward	GTTTTAGAGGAAGGAATGTTGTGAGG
		Reverse	ACAACCCAATATACCACTTCCACCT
59	Chr.10 67595674	Forward	GAAGGTTTGTTGTGTGGAGTTGTAG
		Reverse	ACTTTTAAAACCTTAAATTACAATCTTACCTCA

Additional file 1

Table S4. Chromosomal nucleotide position of methylated cytosine predicted by the Genome DNA Fragment Database (GFDB) and gene names identified by actual sequencing analysis.

Peak ID	Chromosome	Position[a]	Gene name[b]
7	3	83588206	*AC141473.2**
10	9	53410271	*Acat1**
12	4	138720271	down stream both of *Hspe1*and *RP23-2411.9**
26	13	110215936	*Pde4d**
27	2	30409726	9-kbp up stream of *RP23-3990.3**
41	7	31402396	*Cox6b1**
	4	141150829	*Eblim1*
44	7	29587740	3-kbp down stream of *Rin1**
53	5	52443251	100-kbp down stream of *Dxh15**
	19	56431868	*Nrap*
55	11	76224631	*Timm22**
59	10	67595674	*Arid5b**
	7	144033409	50-kbp up stream of *Mgmt*
63	17	44272079	25-kbp up stream of *Enpp4**

[a]Chromosomal position as determined by GFDB.

[b]Name of gene nearest to the target CpG.

*Gene sequences determined by gel-isolation and sequencing analysis that were successfully matched candidate loci predicted by GFDB.

Additional file 1

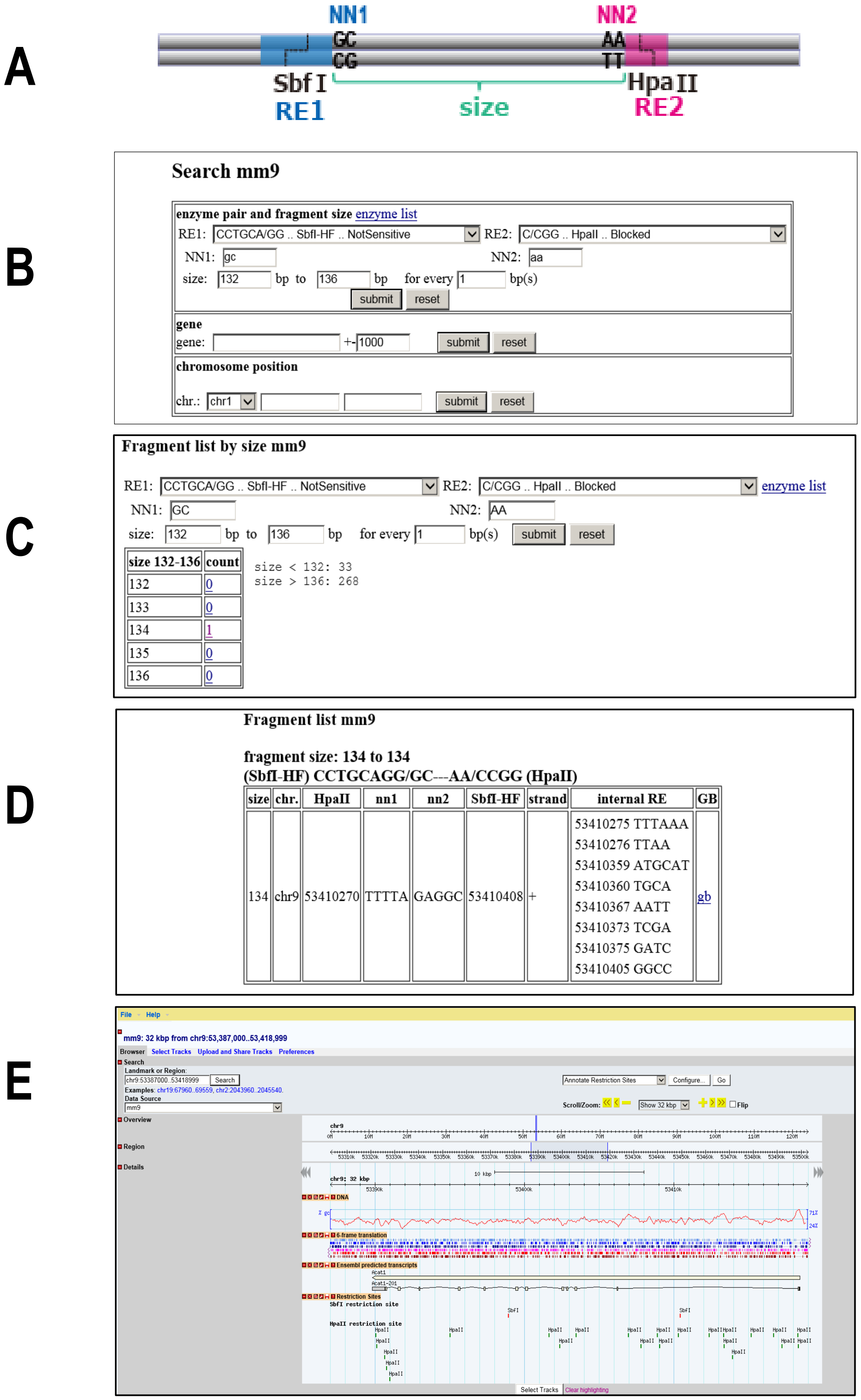

size 132-136	count
132	0
133	0
134	1
135	0
136	0

size	chr.	HpaII	nn1	nn2	SbfI-HF	strand	internal RE	GB
134	chr9	53410270	TTTTA	GAGGC	53410408	+	53410275 TTTAAA<br>53410276 TTAA<br>53410359 ATGCAT<br>53410360 TGCA<br>53410367 AATT<br>53410373 TCGA<br>53410375 GATC<br>53410405 GGCC	gb

Additional file 1

Figure S1. Genome DNA Fragment Database (GFDB). GFDB is a system for predicting the genomic position of CpG sites from AFLP peak charts. Reference genome sequences were from hg19 human database (http://www.ncbi.nlm.nih.gov/assembly/GCF_000001405.13), mm9 mouse databases (http://www.ncbi.nlm.nih.gov/assembly/GCF_000001635.18), dr10 zebrafish databases (https://www.ncbi.nlm.nih.gov/assembly/210611), and nc12 *Neurospora crassa* databases (https://www.ncbi.nlm.nih.gov/assembly/GCF_000182925.2). (**A**) A potential search scenario for fragments, in this example generated by *Sfb*I and *Hpa*II digestions. (**B**) Interface for the selection of restriction enzymes (RE1 and RE2), selective nucleotides (NN1 and NN2), and fragment length range (size). GFDB can simulate the MSD-AFLP process of DNA cleavage with a combination of any restriction enzyme, Pre-PCR reaction, and selective-PCR reaction. (**C**) Display of the number of DNA fragments produced per fragment length by the chosen combination of restriction enzymes and selective nucleotides. (**D**) Display of the genomic location of a methylated *Hpa*II-CpG site, internal sequence of the nearest recognition sequence, and other restriction enzyme sites in the fragment. (**E**) Display of additional genomic information on the genome viewer.

Additional file 1

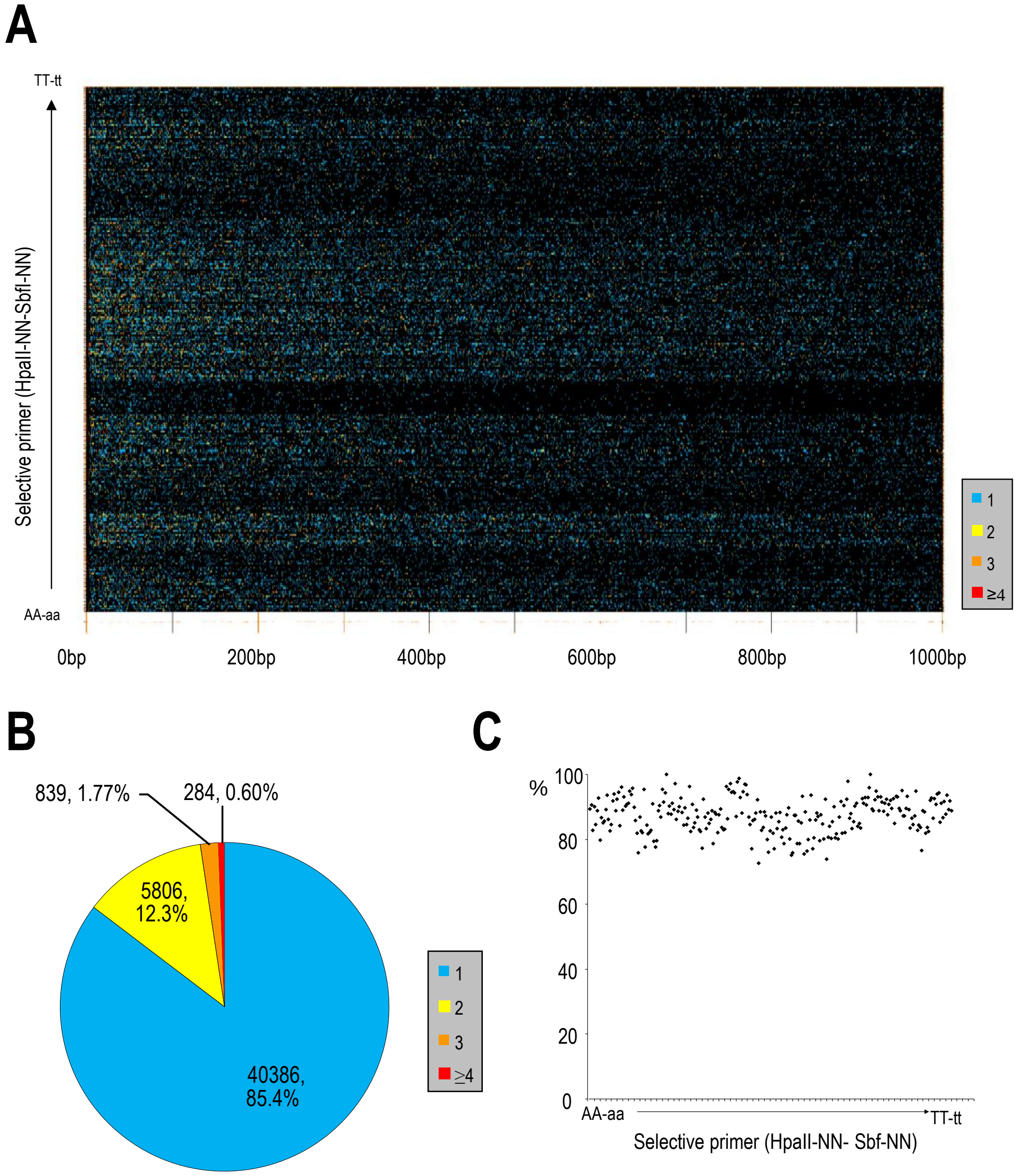

Additional file 1

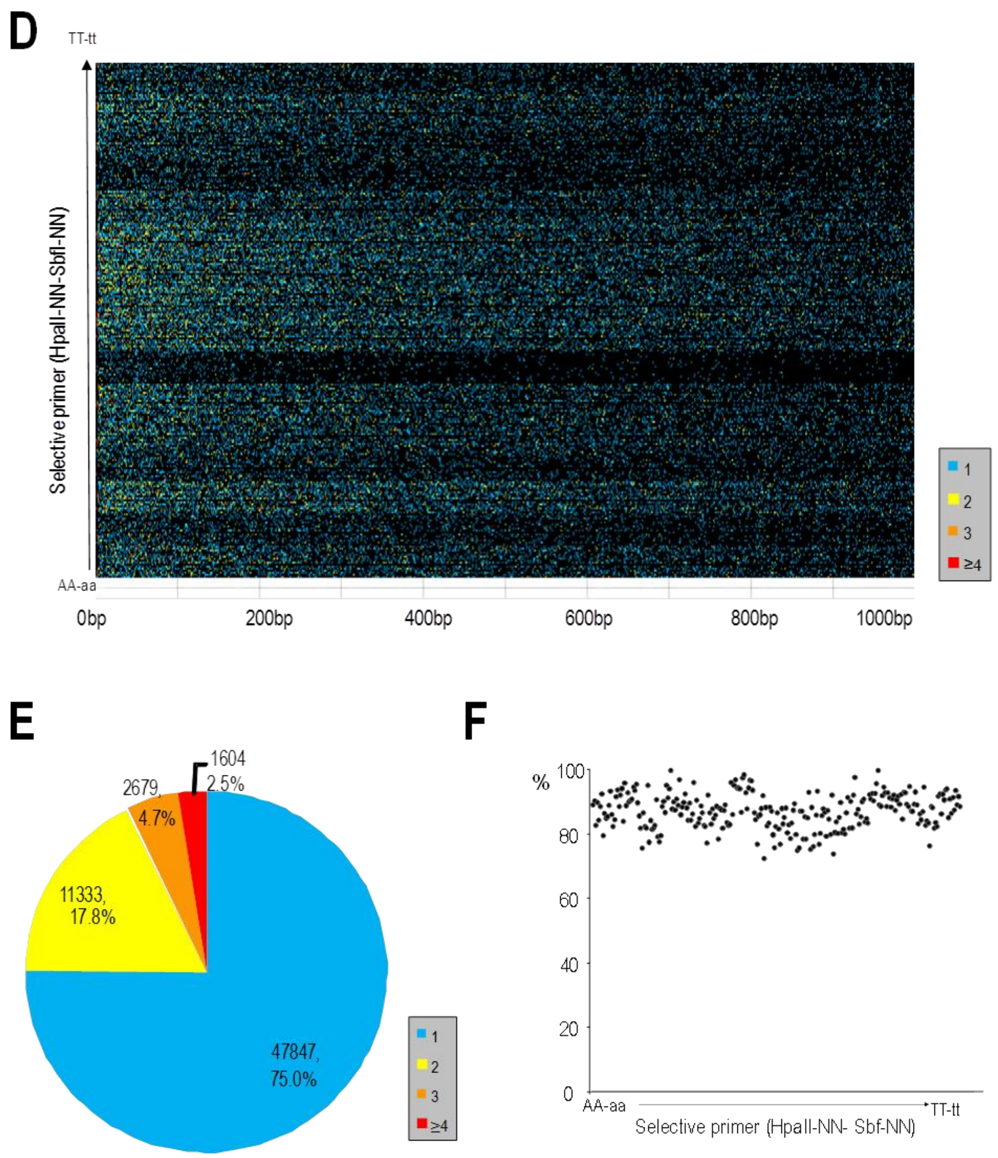

Figure S2. *Sbf*I-*Hpa*II/*Msp*I fragments in the mouse and human reference genome sequence, retrieved using the GFDB system. (**A, D**) The distribution of fragments by fragment length and selective primer in alphabetical order (256 total combinations) in the mouse (**A**) and human (**D**). Blue dots indicate fragment lengths that have only one fragment at that length and so are predicted to display a single peak on an AFLP chart. Yellow, orange, and red dots indicate fragment lengths where two, three, or four or more fragments share the same length, respectively, resulting in difficult to interpret peaks. (**B, E**) Pie chart showing the percentages of the four fragment length distributions in Panel A (**B**) and D (**E**) (blue, one fragment; yellow, two; orange, three; red; four or more). (**C, F**) Percentages of DNA fragment lengths by selective primer combination that have only one fragment at that length in the mouse (**C**) and human (**F**).

Additional file 1

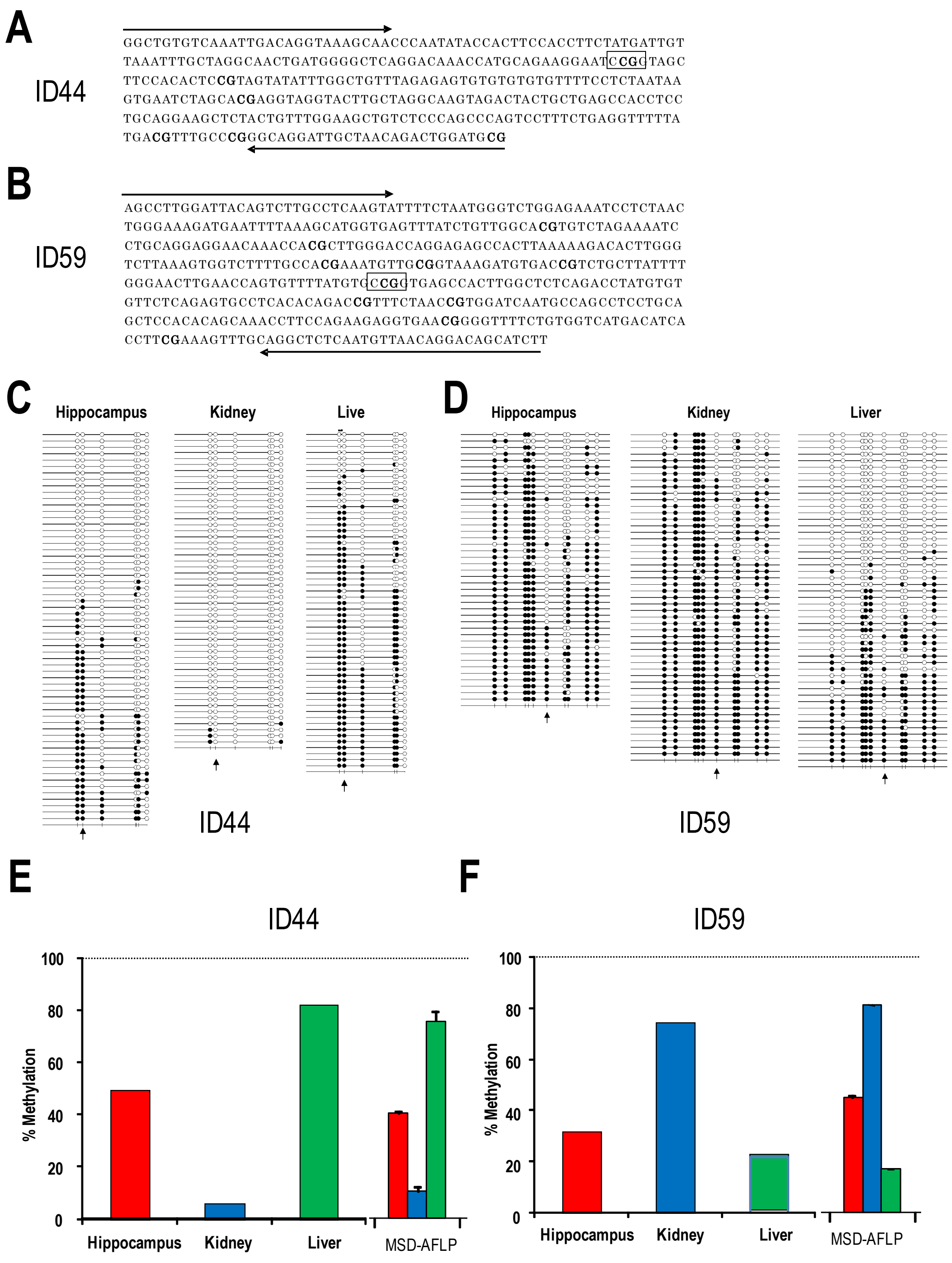

Figure S3. Analysis with bisulfite genome-sequencing. (**A**, **B**) Target regions of the surrounding *Hpa*II site of randomly selected Peak ID 44 and 59. The boxes indicate the *Hpa*II sites. (**C**, **D**) The DNA methylation patterns and frequencies of ID 44 and 59. The target *Hpa*II-CpGs are indicated by arrows. Methylated-CpGs, Black circles; Unmethylated-CpGs, Open circles. (**E**, **F**) The *Hpa*II-CpG percent methylation levels of ID 44 and 59 calculated from Panels C and F, respectively. The percent methylation levels of ID 44 and 59 in each tissue were similar to those calculated by MSD-AFLP (from Figure 5D, represented at right sides in Panels E and F).

Additional file 1

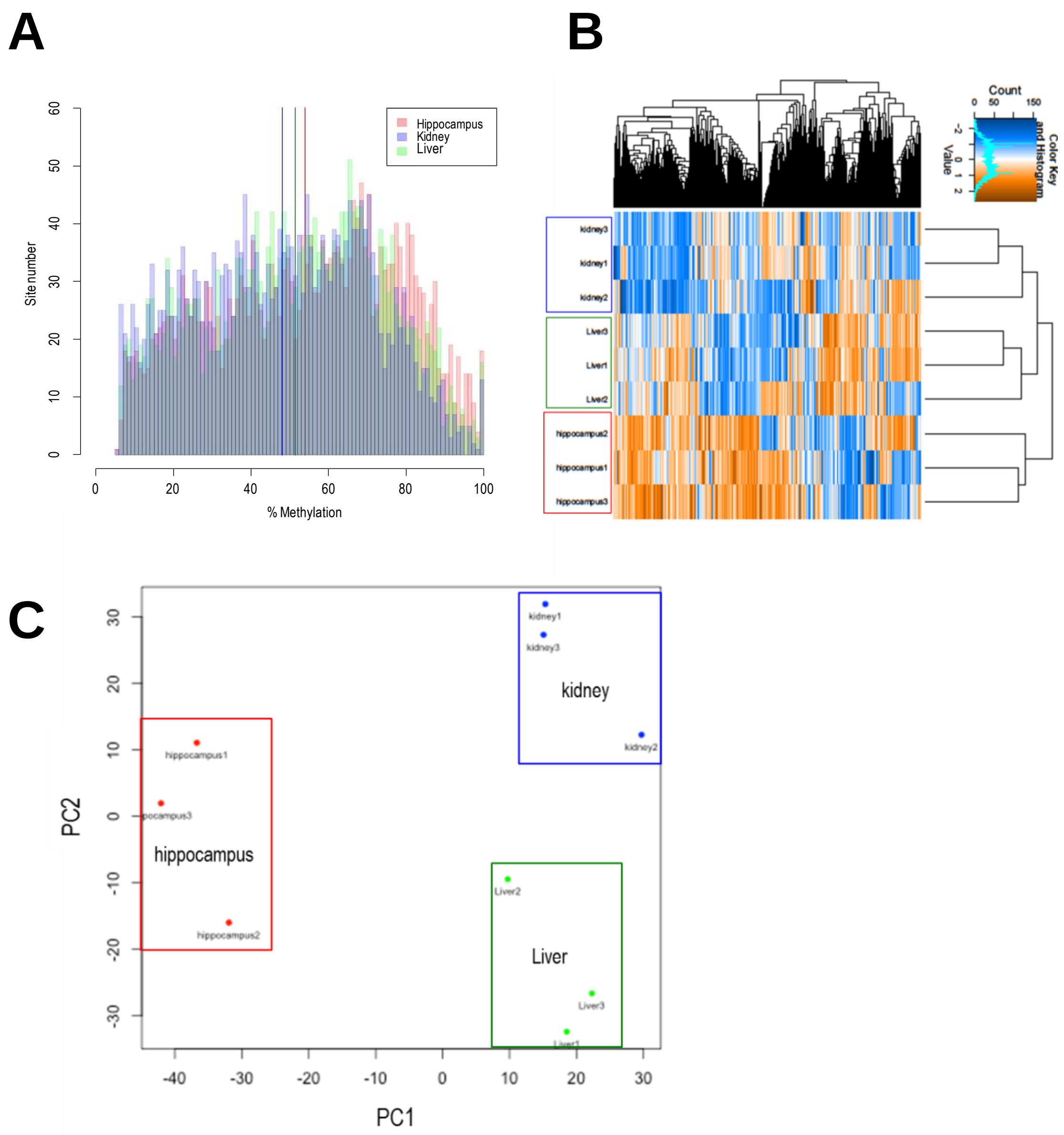

Figure S4. Comparison of genome-wide methylation patterns among tissues using MSD-AFLP data. (**A**) Histogram of the methylation level of all CpGs detected. The red, blue, and green lines indicate the mean percent methylation level of hippocampal (54.1 ± 0.48%), kidney (47.6 ± 0.49%), and liver tissue (51.0 ± 0.46%), respectively. (**B**) Hierarchical clustering analysis of the normalized methylation pattern for each sample utilizing Euclidean distance and Unweighted Pair-Group Method with Arithmetic mean (UPGMA). (**C**) PCA of the normalized methylation pattern for each sample. The red, blue, and green dots and frames indicate hippocampal, kidney and liver samples, respectively.
